# Total body irradiation and pneumonitis risk: a review of outcomes

**DOI:** 10.1038/sj.bjc.6601751

**Published:** 2004-05-04

**Authors:** S A Carruthers, M M Wallington

**Affiliations:** 1 Department of Radiation Oncology, Royal Adelaide Hospital Cancer Centre, North Terrace, Adelaide, South Australia 5000, Australia; 2 Department of Radiation Oncology, Royal Hobart Hospital, The University of Tasmania, Hobart, Tasmania 7000, Australia

**Keywords:** total body irradiation, interstitial pneumonitis

## Abstract

A review was undertaken of all patients treated at Royal Adelaide Hospital, South Australia with total body irradiation (TBI) for the purpose of assessing the incidence of interstitial pneumonitis (IP) and possible prognostic factors for its development. The aim was also to assess the impact of IP and other prognostic factors on long-term survival outcome following bone marrow transplantation. A total of 84 patients received TBI, with 12 Gy in six fractions delivered using two different instantaneous dose rates of 7.5 and 15 cGy min^−1^. This series included 26 cases of acute lymphoblastic leukaemia, 26 of multiple myeloma and 15 of acute myelogenous leukaemia. On multivariate analysis, a higher dose rate was independently significant for an increased risk of IP.

Total body irradiation (TBI) is frequently used for conditioning prior to allogeneic bone marrow transplantation (BMT) due to its immunosuppressive effect on the host immune system, thus minimising the risk of engraftment failure (Torres *et al*, 1982; Cardozo *et al*, 1985; Kader *et al*, 1994). This role has been used principally in acute myelogenous leukaemia (AML) and acute lymphoblastic leukaemia (ALL). In addition to aiding engraftment, TBI provides additional malignant cell kill and is active in chemotherapy inaccessible sanctuary sites. These latter functions are the predominant rationale for the use of TBI in autologous transplantation. Randomised studies of BMT for AML with and without TBI as part of the conditioning regimen have found that TBI regimens provide equivalent or better outcomes for survival (Blaise *et al*, 1992; Blume *et al*, 1993; Dusenbery *et al*, 1995; Hartman *et al*, 1998). Pulmonary complications are a significant source of morbidity and mortality with BMT for haematological malignancies (Tait *et al*, 1991). Interstitial pneumonitis (IP) has a documented incidence ranging from 10 to 84% (Weiner *et al*, 1986). The development of IP is increased by various risk factors including infection, particular types of chemotherapy, the use of single fraction TBI at higher dose rates (Bortin *et al*, 1982; Deeg, 1983), higher total TBI lung dose (Keane *et al*, 1981; Torres *et al*, 1982), methotrexate administration post-transplant (Bortin *et al*, 1982; Weiner *et al*, 1986) and acute graft-versus-host disease (GVHD) (Torres *et al*, 1982; Cardozo *et al*, 1985; Weiner *et al*, 1986; Latini *et al*, 1992; Ozsahin *et al*, 1992, 1994). The relative contributions of each are difficult to establish. The mortality rate with IP has been reported as high as 80% (Gogna *et al*, 1992).

## MATERIALS AND METHODS

A review of all patients treated with TBI at Royal Adelaide Hospital, South Australia revealed a total of 95 patients. Prior to 1982, nine patients were treated with a single dose of 7–8.5 Gy, at 15 cGy min^−1^ prescribed to the mid-pelvis. From 1982 to February 1991, 55 patients received fractionated TBI, with 53 receiving 12 Gy in six fractions over 3 days, with a minimum interfraction interval of 6 h, at an instantaneous dose rate of 15 cGy min^−1^. Two paediatric patients were prescribed fractionated doses of 9 and 8.5 Gy, respectively. From March 1991, the dose rate was reduced to 7.5 cGy min^−1^, with 31 patients receiving 12 Gy in six fractions at this dose rate (Table 1

**Table 1:** Patient characteristics and treatment

Total patients	84
*Sex*	
Male	53
Female	31
Age range (years)	4–66
	
*Diagnosis*	
ALL	26
MM	26
AML	15
NHL	10
Other	7
	
*Dose rate*	
7.5 cGy min^−1^	31
15 cGy min^−1^	53
Mid-pelvis	41
Max lung dose pt.	12
	
*Transplant type*	
Autologous	47
Syngeneic	2
Allogeneic	35

). In order to produce a more homogeneous study population for the purposes of analysis, only those patients who received 12 Gy in six fractions were included, thus excluding nine single dose patients and two lower dose patients. This resulted in 53 patients at 15 cGy min^−1^ dose rate and 31 at 7.5 cGy min^−1^ (total of 84 patients).

The age range of the patients was 4–66 years with a male/female ratio of 1.7 : 1. Primary diagnoses consisted of 26 cases of ALL, 26 of multiple myeloma (MM), 15 of AML, 10 of non-Hodgkin's lymphoma (NHL), and a miscellaneous group including chronic myelogenous leukaemia and Ewing's sarcoma (Table 1). Initial chemotherapeutic management was according to the prevailing best practice guidelines. Many of the cases were enrolled in international and national clinical trials including TBI and BMT. A number of cases were, however, transplanted in relapse or with advanced disease under less than clinically optimal conditions.

*In vivo* dosimetry of prescribed dose was obtained using diode measurements and limited thermoluminescent dosimetry (TLD) measurements prior to 1987 and TLDs at six anatomical sites per patient thereafter (Van Dam and Marinello, 1994). The point of prescribed dose altered over the period of this review, with the dose being prescribed to mid-pelvis between 1982 and 1987. In 1988, computerised tomography (CT)-based computer planning with full lung corrections was implemented, with the point of dose prescription thereafter being the maximum lung dose. For the 15 cGy min^−1^ group, 41 patients (77%) were prescribed to mid-pelvis, 12 (23%) to the maximum lung dose point and all patients in the 7.5 cGy min^−1^ group to the maximum lung dose point. *In vivo* lateral chest wall TLD readings were used to estimate the mean thoracic dose at the level of the second intercostal space. Comparison of readings (*n*=122) for the two prescription methods (mid-pelvis and maximum lung dose point) was performed. All patients had *in vivo* TLD dosimetry during the initial fraction of TBI. In some patients TLDs were repeated during the second fraction for quality assurance purposes. The *in vivo* TLD readings showed a wide range of doses (1.72–2.01 Gy for the mid-pelvis prescription cases, and 1.55–2.26 Gy for the CT planned cases). The mean values were 1.88 and 1.81 Gy, respectively, a difference of 0.07 Gy per 2 Gy fraction. A ‘*t*’-test comparison of the TLD readings for the two prescription point methods did not show any significant difference with a ‘*P*’-value of 0.86 (95% confidence interval – 0.72, 0.86).

All patients were treated with 10 megavoltage (MV) photons with parallel opposed lateral fields at an extended focus to skin distance of 4 m. All patients had chest wall bolus applied for soft-tissue homogeneity and to reduce lung dosage. Lead attenuators were used as needed to compensate for reduced separation in the region of the head and neck, and lower limbs from the mid-thigh down. These attenuators were adjusted in thickness after TLDs when indicated, prior to the third fraction of TBI, to maintain a dose homogeneity within 10% of the prescribed dose. A flat supine position was used for the single fraction patients, with the treatment position changed to a semirecumbent supine one at the time of introduction of fractionated TBI. A 1 cm thick perspex screen adjacent to the treatment couch was used as a beam spoiler to maintain dose to superficial tissues.

Chemotherapy conditioning prior to TBI was generally with high-dose cyclophosphamide (80 of 84 cases). Transplant type following TBI was autologous in 47 cases, allogeneic in 35 and syngeneic in 2. Histocompatibility locus antigen matching was identical in 29 of the 35 cases for allogeneic transplantation. Interstitial pneumonitis was defined as a clinical condition of dyspnoea and hypoxia with bilateral interstitial opacities on chest radiographs (Weiner *et al*, 1986). Infective IP was diagnosed on lung biopsy, sputum culture or serological testing. Histological confirmation (lung biopsy or autopsy) of the clinical diagnosis of IP was possible in only 10 of the 27 cases that developed IP. Acute GVHD was graded 1–4 using standard criteria of severity of organ involvement (Thomas *et al*, 1975). Grades 1–2 included cases with mild-to-moderate skin changes, no or mild gastrointestinal involvement and no or mild decrease in clinical performance, while grades 3–4 had more significant changes.

### Statistical methods

Survival curves were produced using the Kaplan–Meier method with log-rank testing. The 95% CIs for survival time and other study end points are shown within parenthesis. Cox's regression analysis was performed to examine the significance of independent risk factors for survival following TBI. Log binomial regression of prognostic factors for the development of IP was also performed.

## RESULTS

### Survival

**Figure 1:**
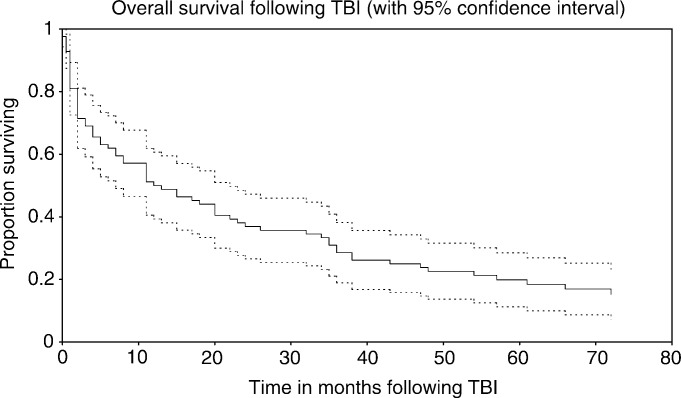
Overall survival following TBI.

Survival, with 95% CIs, for the study cohort as a whole is shown in Figure 1

**Figure 2:**
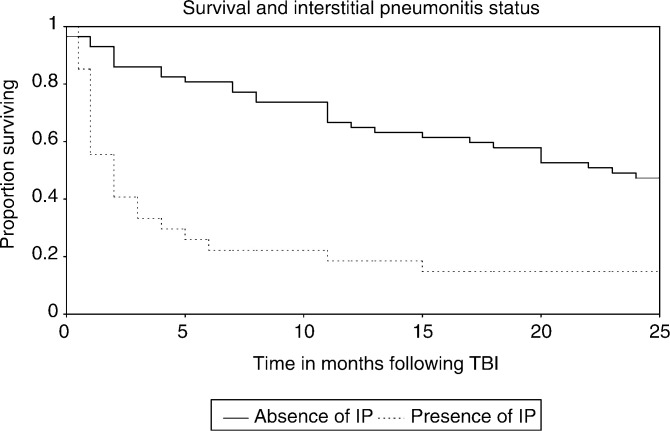
Survival and IP status.

. The overall median survival was 12.5 (7–22) months. Cox's regression analysis of survival found IP (hazard ratio (HR)=2.37 (1.35, 4.15), *P*=0.003) to be independently significant for decreased survival as illustrated in Figure 2

. With respect to disease type, Cox's regression analysis found ALL (*P*=0.002) and the miscellaneous group (*P*=0.01) had statistically significant better survival relative to AML, and NHL (*P*=0.08) was of borderline significance relative to AML. Higher dose rate was significant for poorer survival only on univariate analysis. In all, 14 patients remained alive at the time of analysis, with a total of 116 patient years of observation within this group, and a total of 210 patient years of observation for the entire 84 patients.

### Interstitial pneumonitis

Interstitial pneumonitis developed in 27 cases as shown in Table 2

**Table 2:** Interstitial pneumonitis

Total IP (cases)	27
Idiopathic	17
Infective	10
CMV diagnosed	7
Fatal IP	19
Mortality rate	19/27 (70%)
	
*Dose rate*	
15 cGy min^−1^	23/53 (43%)
Mid-pelvis	16/41 (39%)
Max lung dose pt	7/12 (58%)
7.5 cGy min^−1^	4/31 (13%)

IP=Interstitial pneumonitis.

, with infective causes in 10 patients in whom seven cases of cytomegalovirus were detected. The analysis looked at all cases of IP regardless of the presence of infection or not (idiopathic IP), with a median time to onset of IP post-TBI of 30 days (range, 10–560 days), with two cases beyond 6 months, both idiopathic and associated with lung GVHD, with one case fatal at 14 months. The mortality rate for IP was 70% (19 of 27) patients. Acute GVHD developed in 23 of 35 (66%) allogeneic patients, with grade 1–2 in 15 patients and grade 3–4 in eight patients.

Table 3

**Table 3:** Univariate log binomial regression of risk factors for IP

	**At risk**	**Developed IP (%)**	**Unadjusted relative risk**	**95% CI**	***P*-value**
Age (years)			0.98	(0.97, 1.00)	0.08
*Sex*					
Female	31	12 (39%)	1.00		
Male	53	15 (28%)	0.73	(0.39, 1.35)	0.32
					
*Transplant type*					
Autologous/other	49	10 (20%)	1.00		
Allogeneic	35	17 (49%)	2.38	(1.24, 4.56)	0.01
					
*Dose rate*					
7.5 cGy	31	4 (13%)	1.00		
15 cGy	53	23 (43%)	3.36	(1.28, 8.83)	0.004
					
*GVHD*					
Absent	12	4 (33%)	1.00		
Present	23	13 (57%)	1.70	(0.71, 4.07)	0.19
					
*GVHD*					
Grades 1–2	15	9 (60%)	1.00		
Grades 3–4	8	4 (50%)	0.83	(0.37, 1.87)	0.65

IF=Interstitial pneumonitis; CI=confidence interval.

shows the univariate log binomial regression analysis of prognostic risk factors for IP including age, diagnosis, sex, transplant type, dose rate, GVHD and grade of GVHD. Higher dose rate and allogeneic transplantation were found to be significant for a higher risk of IP.

Using log binomial regression, only dose rate was found to be independently significant for the development of IP, with a dose rate of 15 cGy min^−1^ (RR=3.00 (1.06, 8.43), *P*=0.04) significantly increasing the risk of IP. At 7.5 cGy min^−1^, there were four of 31 (13%) cases of IP, while at 15 cGy min^−1^, 23 of 53 (43%) cases were described following BMT. In terms of the dose prescription method, there were 16 of 41 (39%) cases at 15 cGy min^−1^ to the mid-pelvis, seven of 12 (58%) cases at 15 cGy min^−1^ to the maximum lung dose point, with four of 31 (13%) cases at 7.5 cGy min^−1^ (all to the maximum lung dose point). The incidence of idiopathic IP alone with respect to dose rate shows one case only of 31 (3%) at 7.5 cGy min^−1^ and 16 of 53 (30%) cases at 15 cGy min^−1^. The incidence of IP for allogeneic transplantation was 17 of 35 (49%) cases and for autologous 10 of 49 (20%) cases. Of 35 allogeneic transplant patients, four (33%) cases of IP were found among 12 patients without GVHD and 13 (57%) cases among 23 patients with GVHD.

## DISCUSSION

We have found an association between dose rate and risk of IP in this review of TBI. In previously published data on dose rate and the risk of IP, a randomised controlled trial of fractionated TBI assessing the influence of dose rate on the incidence of pneumonitis found no significant difference for lower range dose rates of 3 and 6 cGy min^−1^, with lung shielding limiting the total lung dose to 9 Gy (Ozsahin *et al*, 1992). Similarly, a retrospective analysis did not find a dose rate effect comparing dose rates of 2.9–6.5 cGy min^−1^ with 6.9–8.9 cGy min^−1^ for fractionated TBI (Gogna *et al*, 1992), but again this study used lower dose rates than in our study. Other publications have stated that with fractionated doses of 200 cGy, dose rate is of little importance for most tissues since cell death results predominantly from nonrepairable single hit killing (Peters *et al*, 1979; Peters, 1980).

In contrast, one univariate analysis found a dose rate >6 cGy min^−1^ correlated with an increased risk of IP for fractionated TBI at 2 Gy per fraction (Corvo *et al*, 1999). A large retrospective review of 932 leukaemic patients who were managed with allogeneic BMT found a dose rate of less than 6 cGy min^−1^ was associated with a decreased incidence of IP, with the dose rate effect significant for those patients who had received methotrexate prophylaxis for GVHD (Weiner *et al*, 1986). Two retrospective analyses that found decreased incidence rates of IP at dose rates of <5.8 and 2.5 cGy min^−1^, respectively, involved patients treated with a single fraction of TBI where a dose rate effect may be expected to be present (Bortin *et al*, 1982; Barrett *et al*, 1983).

Support for a possible dose rate effect can also be found in experimental animal data where the lethal dose for 50% mortality (LD_50_) from pneumonitis increases with decreasing dose per fraction below 2 Gy per fraction, suggesting normal tissue repair at doses below 2 Gy, and hence increased repair at decreased dose rates for 2 Gy fractions (Parkins *et al*, 1985).

The analysis as in our series of all cases of IP without separation of infective from idiopathic cases has been performed previously (Bortin *et al*, 1982; Weiner *et al*, 1986; Ozsahin *et al*, 1992; Girinsky *et al*, 1994; Morgan *et al*, 1996). We have chosen not to analyse separately as the methods of diagnosing infection are not equivalent for all the cases, such that a proportion of infective cases from the earlier years may have been classified erroneously as idiopathic. Moreover, if it is argued that it is only valid to analyse idiopathic cases since infective IP may be due to a number of causes other than TBI, then the crude incidence figures for our series for idiopathic IP demonstrate a strong dose rate relationship, with only one case of 31 at 7.5 cGy min^−1^ and 16 of 53 (30%) cases at 15 cGy min^−1^.

With respect to transplant type, other authors have demonstrated a reduced risk of IP with autologous transplantation. One analysis found the risk of IP to be 27% (27 of 99) with allogeneic transplantation and 0% (zero of nine) with autologous, but this was not statistically significant due to low numbers (Morgan *et al*, 1996), while other literature reports a significantly decreased incidence of IP after autologous transplantation compared to similarly treated allogeneic transplant patients (Wingard *et al*, 1998). We were only able to demonstrate, however, a reduced risk of IP for autologous transplantation on univariate analysis.

Our study has not found acute GVHD to be an independent prognostic factor for IP. Previous literature has reported the absence of GVHD as contributing to a reduced risk of IP (Latini *et al*, 1992), and the presence of severe GVHD to be a significant risk factor for IP and early mortality (Weiner *et al*, 1986; Valls *et al*, 1993). Graft-versus-host disease was found on multivariate analysis of a randomised study to be significant for an increased risk of IP (Ozsahin *et al*, 1992), and a similar result was found in an earlier analysis (Torres *et al*, 1982). Conversely, two retrospective studies with mostly fractionated TBI could find no correlation between acute GVHD and the incidence of IP (Morgan *et al*, 1996; Aristei *et al*, 1998).

Our statistical analysis excluded single fraction patients, but it is worth noting that on review, six of our nine single dose patients developed IP. Fractionation as a factor for a reduced risk of IP is not always consistently reported in the literature, where the single doses tend to be lower, and hence are associated with an increased risk of leukaemic recurrence (Girinsky *et al*, 1994). One early randomised trial comparing a single dose of 10 Gy with fractionated TBI of 12 Gy in six fractions found no significant difference for the incidence of IP (Thomas *et al*, 1982). A nonrandomised study assessed the influence of fractionation on the incidence of pneumonitis, finding no difference between single fraction and fractionated treatment (Kim *et al*, 1990), but this study used a modified single fraction TBI schedule with a total dose of only 7.5 Gy (Cosset *et al*, 1994). Other retrospective studies have shown reduced risks of IP and fatal IP with fractionated TBI (Socie *et al*, 1991; Sutton *et al*, 1993). Hyperfractionated TBI schedules have found much reduced incidences of IP compared to single doses and standard fractionated doses (Cosset *et al*, 1989; Latini *et al*, 1992). The common feature of the above reviews of IP incidence and fractionation is that higher total doses are possible with fractionation, but the lungs remain the dose-limiting normal tissue for TBI.

In further considering the significance of dose rate as an important determinant of outcome following TBI, the possibility that the lower dose rate was linked in our cases to the benefits of CT-based planning allowing the lung tolerance bar to be set more accurately must be acknowledged. Nevertheless, for patients prescribed to the maximum lung dose point through CT-based planning, there is a large difference in IP incidence of seven of 12 (58%) and four of 31 (13%) cases for 15 and 7.5 cGy min^−1^, respectively. While a small mean difference of 3.5% (0.07 Gy) in dose/fraction, as observed within the wide dose ranges recorded on TLDs for the two prescription methods groups, was not statistically significant, there is a potentially slightly higher dose for that proportion of the 15 cGy min^−1^ patients prescribed to mid-pelvis. However, the IP incidence figures show 16 of 41 (39%) IP cases for 15 cGy min^−1^ to mid-pelvis, and a higher incidence of IP of seven of 12 (58%) cases for 15 cGy min^−1^ to the maximum lung dose point.

Finally, the possibility of other time-dependent improvements in patient management, as confounding factors, cannot be excluded in considering the results of our review. Such improvements include improved bone marrow harvesting and storage, more efficacious antibiotics post-transplant, greater use of cytokines to recover pancytopenia and general improvements in clincal diagnosis and care, all of which may have significantly contributed to the reduced risk of IP in the latter cohort treated at 7.5 cGy min^−1^.

In conclusion, this review of TBI conditioning for BMT provides a more mature long-term analysis of outcomes than other reviews in the literature. A higher TBI dose rate has been shown to be an adverse prognostic factor for developing IP, confirming earlier data from a number of centres (Bortin *et al*, 1982; Barrett *et al*, 1983; Weiner *et al*, 1986; Morgan *et al*, 1996; Wingard *et al*, 1998; Corvo *et al*, 1999). As long-term survival is possible following BMT for those who survive the early post-transplant dangers of IP, the use of fractionated TBI at a dose rate of 7.5 cGy min^−1^ or less rather than 15cGy min^−1^ is recommended, as a means of reducing IP and increasing survival in cases where such an option exists. The importance of high-level quality assurance procedures and CT-based lung corrected dosimetry for TBI is also emphasised.
